# The effect of enhanced structure in the posterior segment of clear aligners during anterior retraction: a three-dimensional finite element and experimental model analysis

**DOI:** 10.1186/s40510-023-00502-2

**Published:** 2024-01-15

**Authors:** Xiaohan Jin, Xue Tian, Victoria Lee Zhi Hui, Yikan Zheng, Jinlin Song, Xianglong Han

**Affiliations:** 1 State Key Laboratory of Oral Diseases, National Clinical Research Center for Oral Diseases, Department of Orthodontics, West China Hospital of Stomatology, Sichuan University, 14# 3Rd Section of Ren Min Nan Road, Chengdu, 610041 Sichuan Province China; 2 Jiangmen Municipal Stomatology Hospital, Yongli Street, Shengli Road, Jiangmen, 529000 Guangdong Province China; 3 R&D Center, EA Medical Center Device Technologies Co., Ltd, Middle Branch Road, Wuxi, 214174 Jiangsu Province China; 4 Chongqing Key Laboratory of Oral Diseases and Biomedical Sciences, Chongqing Municipal Key Laboratory of Oral Biomedical Engineering of Higher Education, Stomatological Hospital of Chongqing Medical University, Chongqing Medical University, 426# Songshibei Road, Chongqing, China

**Keywords:** Clear aligner, Extraction case, Anterior retraction, Finite element analysis, Anchorage reinforcement

## Abstract

**Background:**

Mesial tipping of posterior teeth occurs frequently during space closure with clear aligners (CAs). In this study, we proposed a new modification of CA by localized thickening of the aligner to form the enhanced structure and investigate its biomechanical effect during anterior retraction.

**Methods:**

Two methods were employed in this study. First, a finite element (FE) model was constructed, which included alveolar bone, the first premolars extracted maxillary dentition, periodontal ligaments (PDL), attachments and aligners. The second method involved an experimental model—a measuring device using multi-axis transducers and vacuum thermoforming aligners. Two groups were formed: (1) The control group used common CAs and (2) the enhanced structure group used partially thickened CAs.

**Results:**

FE model revealed that the enhanced structure improved the biomechanics during anterior retraction. Specifically, the second premolar, which had a smaller PDL area, experienced a smaller protraction force and moment, making it less likely to tip mesially. In the same vein, the molars could resist movement due to their larger PDL area even though they were applied larger forces. The resultant force of the posterior tooth was closer to the center of resistance, reducing the tipping moment. The canine was applied a larger retraction force and moment, resulting in sufficient retraction of anterior teeth. The experimental model demonstrated a similar trend in force variation as the FE model.

**Conclusions:**

Enhanced structure allowed force distribution more in accordance with optimal principles of biomechanics during the extraction space closure while permitting less mesial tipping and anchorage loss of posterior teeth and better retraction of anterior teeth. Thus, enhanced structure alleviated the roller coaster effect associated with extraction cases and offered a new possibility for anchorage reinforcement in clear aligner therapy.

**Supplementary Information:**

The online version contains supplementary material available at 10.1186/s40510-023-00502-2.

## Background

Nowadays, clear aligners (CAs) have gained widespread acceptance for treating malocclusion due to their aesthetic and comfortable features [1, 2]. However, long-distance space closure in cases of premolar extraction with aligners can be challenging for orthodontists, owing to the occurrence of the “roller coaster” effect. This phenomenon involves the loss of anchorage, resulting in the distal tipping of anterior teeth and mesial tipping of posterior teeth [3]. While poor patient compliance and protocol design issues can contribute to this phenomenon [4, 5], the more crucial factors are insufficient material stiffness and uncertain biomechanical mechanisms [3, 6]. The stiffness of CA material is equivalent to, or even less than, the nickel–titanium archwire in fixed appliances [7–9]. As a result, flexible aligner material is not suitable for teeth movement at all stages due to their susceptibility to deformation, especially for space closure. Additionally, CAs wrap around the entire crown and apply forces to multiple points or surfaces of the teeth, making the biomechanical mechanism more complex than that of fixed appliances [6]. Therefore, reinforcing the anchorage of posterior teeth and clarifying its biomechanical mechanisms during space closure is a critical clinical problem that must be addressed in clear aligner technology.

In recent years, scientists and clinicians have conducted numerous studies attempting to address this issue by means of protocol design, ancillary devices and material modifications. One approach is to consider anchorage preparation of posterior teeth [10], as well as torque compensation of the anterior teeth [11] during the design phase for cases involving extraction. While helpful, this approach causes reciprocal movement of teeth, which may burden periodontal tissue. Furthermore, the value of this anchorage design lacks standardization, and achieving the desired outcome is uncertain due to various influencing factors in clear aligner tooth movement. Auxiliary appliances include attachments, power arms and mini-implants. The use of ancillary devices can increase the complexity of clinical operations and chairside time. Besides, excessive use of attachments increases the difficulty of aligner insertion and removal. The power arm, while capable of controlling the point of force application closer to the center of resistance (CR), has a strong foreign body sensation and poor esthetics. Moreover, neither the attachments nor the power arms can be bonded to veneers and crowns. Although mini-implants can reinforce posterior tooth anchorage, there are multiple complications of implant fracture, implant loosening, soft tissue inflammation and root contact. Additionally, attempts to improve material properties of the clear aligner diaphragm through blending modification [12] and multi-layer structures [13] have had some success in enhancing force delivery and decreasing stress relaxation, but balancing stiffness and resilience remains a challenge. Furthermore, overall diaphragm material change cannot alter local stress distribution in the posterior area, so the mesial tipping of posterior teeth cannot be prevented precisely. As a result, to date, there has not been a satisfactory method to minimize anchorage loss of posterior teeth during the extraction space closure.

Studies have demonstrated a strong correlation between aligner thickness and the forces delivered by it, indicating that thicker diaphragms transmit greater forces [14, 15]. However, increasing overall thickness changes the mechanical properties of the entire aligner and fails to address local stress distribution in the posterior teeth region. What is more, the process of vacuum thermoforming aligners creates an uneven thickness of the aligner [16, 17]; thus, the thickness of the diaphragm is not indicative of the thickness of the posterior part. Uneven thickness of thermoformed aligners has an impact on the stress distribution [15]. Closing the extraction space with CAs is akin to closing it on a nickel–titanium archwire, which is too flexible and contributes to the “roller coaster” effect. Ideally, the posterior segment of the archwire should be more rigid than the anterior segment [18, 19], so that the tipping of anchor teeth during space closure could be well avoided. Building on this idea, we hypothesize that by thickening the local thickness of the posterior region of CAs to improve the stiffness, the force distribution of the posterior teeth during the anterior retraction could be changed so that the mesial tipping of the posterior teeth would be reduced. Through a three-dimensional finite element (FE) model and an experimental model, we investigated the biomechanical changes resulting from partially thickened CAs, providing new insights for advancing clear aligner therapy.

## Materials and methods

### Finite element model study

**Fig. 1:**
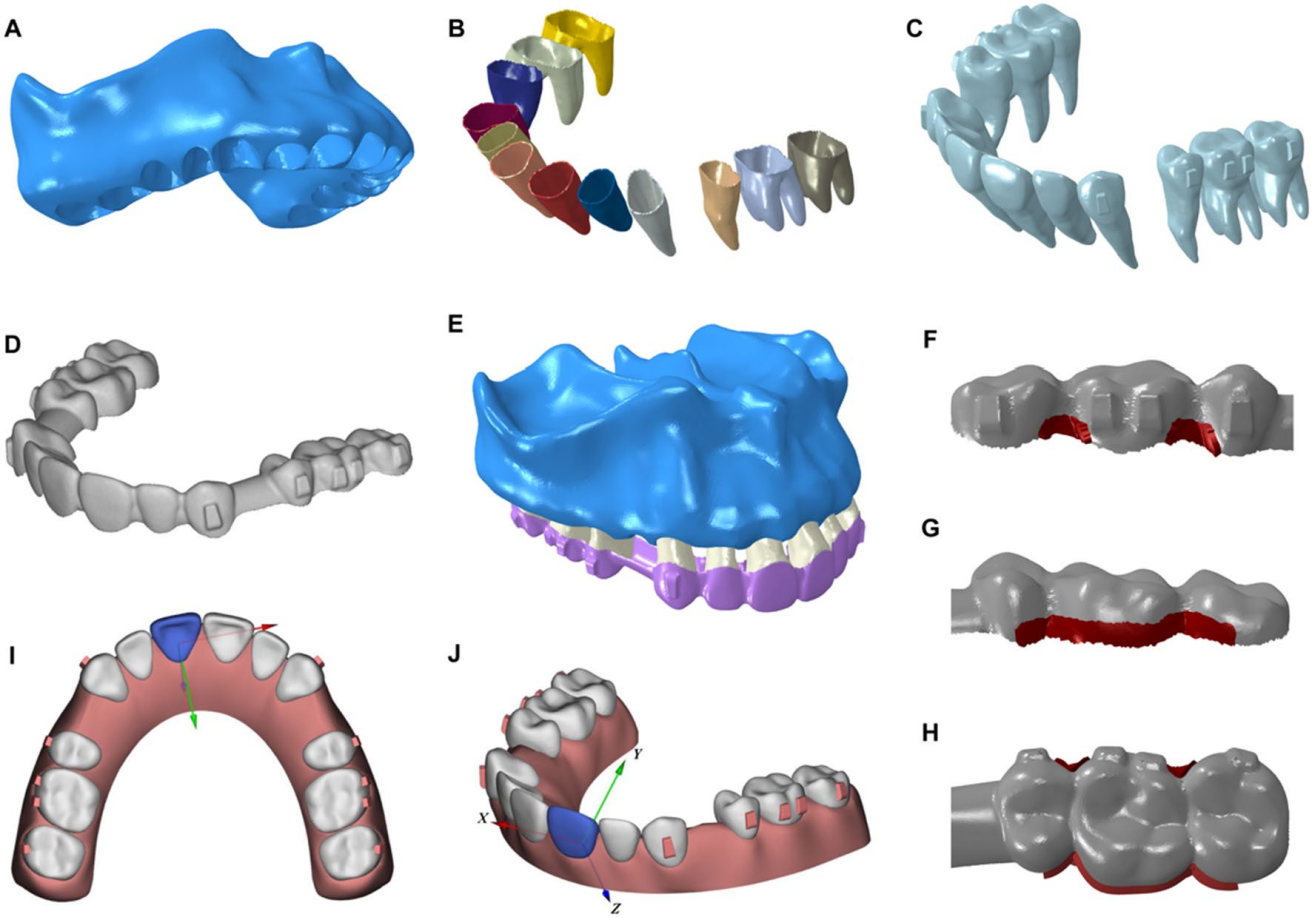
Three-dimensional finite element model: **A**, Alveolar bone; **B**, Periodontal ligament; **C**, Maxillary dentition without the first premolars; **D**, Clear aligner; **E**, The assembled research model; **F–H**, Clear aligner with the enhanced structure in different views; **I-J**, Local coordinate system for each tooth

A healthy adult with well-aligned complete dentition was selected as the subject for this study. Based on cone-beam computed tomography (CBCT) data, the three-dimensional model of alveolar bone and the maxillary dentition with the extraction of the first premolars were reconstructed using Mimics Research 21.0 (Materialize, Leuven, Belgium) and Geomagic Studio 2016 (3D systems, Rock Hill, SC, USA). The periodontal ligament (PDL) was obtained by making an external offset from the root surface, and the thickness was set at 0.3mm [20]. Vertical rectangular attachments (3mm height, 2mm width, 1mm thickness) were set on the buccal surface of canines, the second premolars and molars. The crowns and attachments were extended outward to model CAs with a uniform thickness of 0.75 mm. All the components were meshed with HyperMesh 14.0 (Altair, Troy, Mich, USA) and then imported into ABAQUS 2016 (Dassault SIMULIA, Providence, RI, USA) to be assembled into the three-dimensional finite element model. As shown in Fig. 1 A-E, the FE model consisted of alveolar bone, PDL, teeth with attachments and CA. The number of nodes and elements for each component of the model are shown in Table 1. All the components were regarded as linear elastic, isotropic and homogeneous materials in our study based on previous studies [21, 22]. The mechanical properties of all components are shown in Table 2. The properties of teeth, attachments, PDL and alveolar bone were obtained from the literature [23–26], and the properties of CA was provided by Angelalign Inc.

**Table 1:** Number of nodes and elements of all components in finite element model

Component	Element type	Elements	Nodes
Teeth and Attachments	C3D4	161,304	80,751
PDL	C3D6	85,082	43,342
Clear aligner	S3	72,914	73,926
Enhanced structure	S3R	2948	1794
Alveolar bone	C3D4	159,890	79,947

**Table 2:** Material properties of all components in finite element model

Component	Young’s Modulus (MPa)	Poisson’s Ratio
Teeth and attachments	20,700	0.3
PDL	0.47	0.45
Clear aligner	1000	0.4
Enhanced structure	2000	0.4
Alveolar bone	13,700	0.3

In this study, we proposed a new aligner scheme by thickening the marginal region of the aligner with a width of 1.5 mm by an additional 0.5 mm, hence creating an “enhanced structure.” The FE model was divided into a control group without the enhanced structure and an enhanced structure group. The parameters and properties of the enhanced structure are shown in Tables 1 and 2. In a preliminary study, different variations of enhanced structures were tested, including buccal interproximal space between the posterior teeth (Additional file 1: Fig. 1), palatal cervical line of posterior teeth (Additional file 1: Fig. 2) and both the buccal and palatal sides of the posterior teeth (Fig. 1 F–H). The results (Additional file 1: Table 1–3) indicated that the enhanced structure on both buccal and palatal sides was most effective; and therefore, this type of enhanced structure was selected for further analysis.

The interactions of these parts in FE model were treated carefully to simulate the real situation. The external surface of each tooth’s PDL was connected directly to the corresponding alveoli through the same nodes in the FE model, so it is between the inner surface of PDL and the root of the tooth. Fixed boundary condition was set to the upper transverse section of the maxillary bone. The designed treatment plan involved 0.3mm retraction of anterior teeth without torque compensation of the anterior teeth and anchorage preparation of the posterior teeth. The aligner with designed teeth movement was assembled on the dentition and their interaction was simulated by numerical algorithm of surface-to-surface contact with a friction coefficient of 0.3. The interaction effects, including the deformation and stress of the aligner and the force and moment generated by the aligner and acting on the teeth were solved by the finite element analysis synchronously.

A local coordinate system (LCSYS) was established for each tooth, represented by the X, Y, and Z axes (Fig. 1I-J). The X-axis represented the mesial-distal direction, with the mesial direction being positive. The Y-axis represented the buccal-lingual direction, with the lingual orientation being positive. The Z-axis represented the long-axis direction of the tooth, with the positive direction toward the apex. The initial forces (F, g) and moments (M, gmm) components in the LCSYS of each tooth were measured, referring to CR, and the distance (d, mm) was calculated by dividing M by F. To prevent repetition, only the right side of the maxillary dentition was presented here, as the biomechanical situation on both sides of the dentition was essentially symmetrical and the results on the left side were similar to those on the right side. Von Mises stress distribution of clear aligner and force distribution of maxillary dentition were analyzed.

### Experimental study

**Fig. 2:**
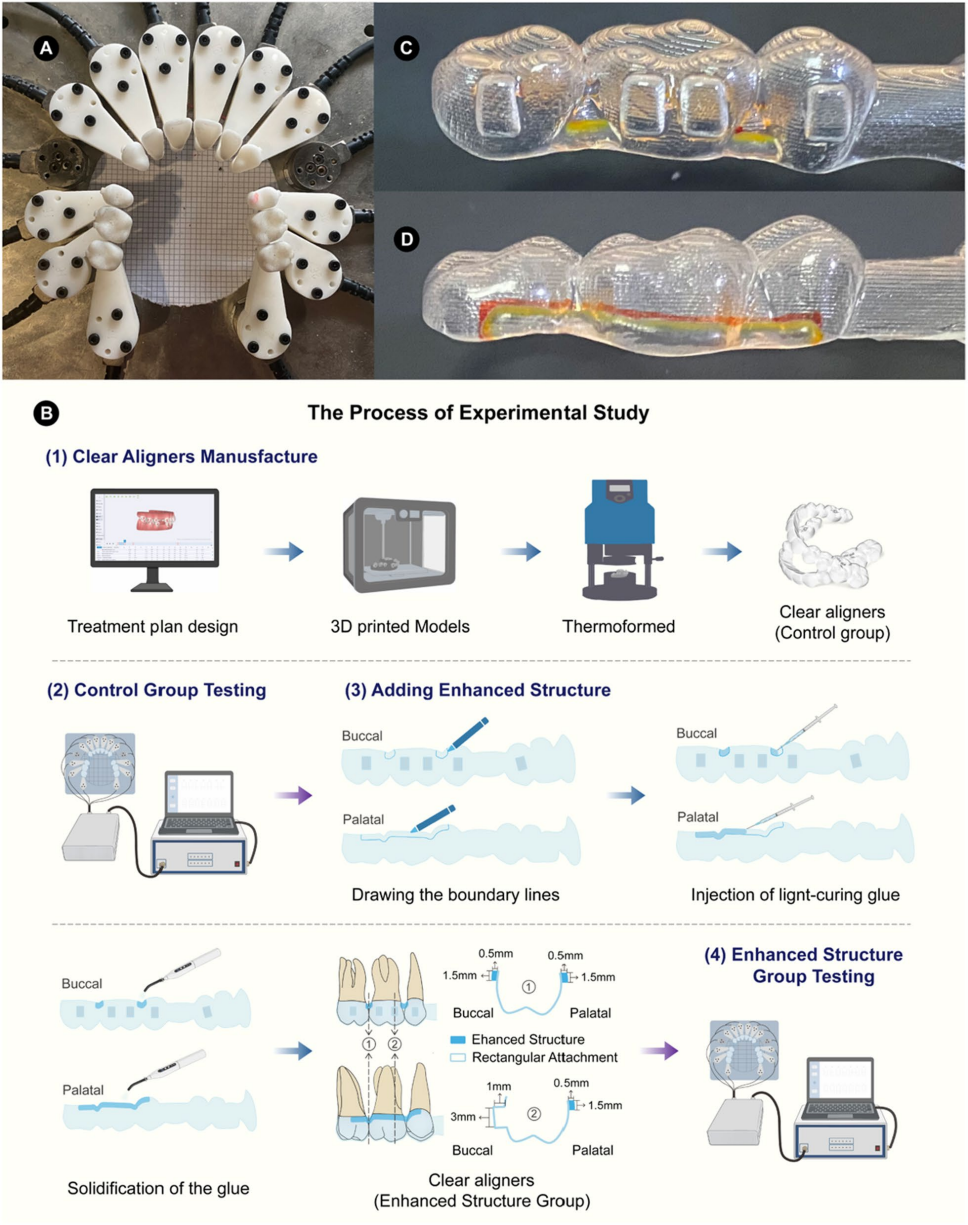
Experimental model: **A**, Mechanical testing device; **B**, The process of experimental study; **C-D**, Clear aligner with the enhanced structure in different views

The physical experiments were conducted with a specially developed mechanical testing device, which can measure the forces and moments in the three-dimensional direction of each tooth [27–30]. This device consisted of 12 high-precision Force/Torque sensors (Nano 17-E, ATI Industrial Automation, Apex, NC, USA), 12 isolated 3D-printed resin teeth (Object 30 OrthoDesk, Stratasys Ltd, MN, USA), and a set of data acquisition and processing software (Fig. 2A). Each resin tooth of the maxillary dentition was separately attached to a sensor using three fixed screws.

The method of the experimental study is presented in Fig. 2B. The designed treatment plan was the same as the FE model. The maxillary dentition from the FE model was 3D-printed (Objet30 Pro, Objet Ltd., Rehovot, Israel) for the production of the corresponding CA for the control group by thermoforming with 0.75 mm diaphragms (Young’s Modulus 1000 MPa, Angelalign Technology Inc., Shanghai, China). Each of the six CAs of the control group was then worn on the experimental device, and the initial force and moment in the three-dimensional direction were collected as the results of the control group. After completing the testing, each of the six CAs in the control group required further processing to create aligners with enhanced structures. The dimensions of the enhanced structure of the experimental model were identical to those of the FE model simulation. For the enhanced structure group in the experimental study, the margins of the thickened area were initially drawn on both the buccal and lingual sides of the aligner at the posterior teeth to determine the length and width. The length was determined separately on the buccal and palatal sides according to the anatomical features, and the width was 1.5 mm for both. Subsequently, a syringe was used to uniformly injected light-curing glue (Angelalign Technology Inc., Shanghai, China) with Young’s modulus of 1980 MPa within the thickened region and then irradiated for 10 s with a dental LED curing light to form an enhanced structure with a thickness of 0.5 mm (Fig. 2C-D). The fabricated enhanced structures were measured to verify their dimensions as described in the Additional file 1: Fig. 3. Finally, the enhanced structure group was measured for forces and moments following the same approach as the control group.

The LCSYS of each tooth was defined in the same way as in the finite element model study. The initial forces (F) and moments (M) components in the LCSYS of each tooth were measured six times. The Shapiro–Wilk test was used to verify the normal distribution of data. Statistical analysis was performed using paired t tests or Wilcoxon signed-rank test for F and M in GraphPad Prism 9.0 (Dotmatics, USA). P < 0.05 was considered statistically significant.

## Results

### F, M and d in the mesial-distal direction of the maxillary dentition

During the retraction of anterior teeth, all F and M values of different directions recorded by both methods were reported in the supplementary information (Additional file 1: 3–6). When closing the extraction space of the first premolar, the posterior teeth are primarily applied forces and moments in the mesial-distal directions.

**Fig. 3:**
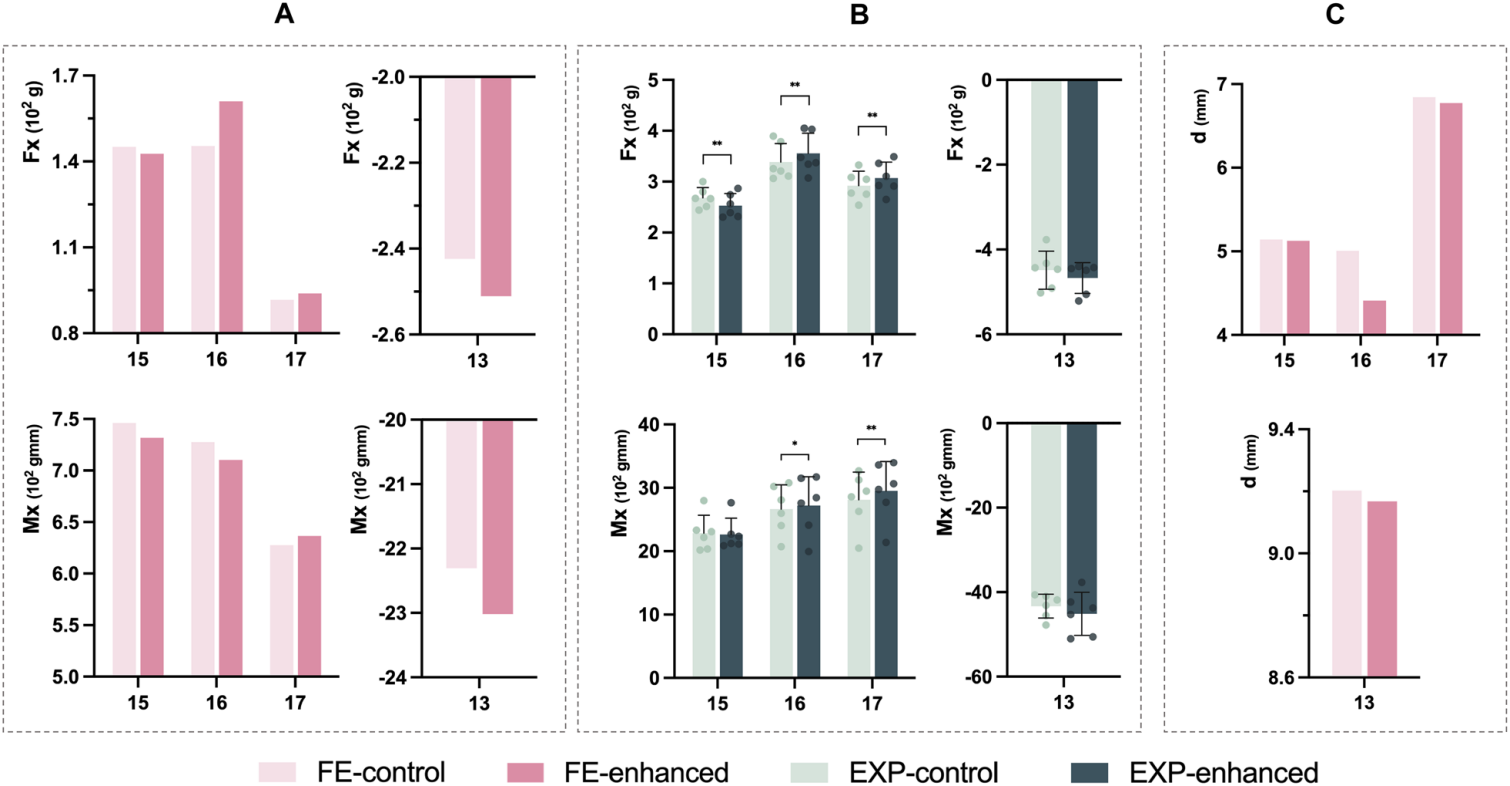
F, M and d in the mesial-distal direction of the maxillary dentition: **A**, Fx and Mx of the finite element model; **B**, Fx and Mx of the experimental model (*P < 0.05, **P < 0.01); **C**, d of the finite element model

As shown in Fig. 3, the second premolar had positive F, M and d values and oriented toward the mesial. In the finite element simulation, the magnitudes of Fx and Mx decreased in the enhanced structure group compared to the control group, indicating a downward trend in mesial tipping movement of the second premolar. The decline of d in the enhanced structure group demonstrated that the resultant force was closer to the CR. In the experimental study, the decreasing trend of Fx magnitude was more noticeable (*P* < 0.01, 95%CI -26.38 to -10.92), but the drop in Mx values was not statistically significant (*P*  > 0.05, 95%CI -106.00 to 65.75).

Both the first and second molars were applied mesial forces during the anterior retraction. In both the FE model and the experimental model, the Fx values for the molars in the enhanced structure group were higher than those in the control group and showed statistical significance (*P*  < 0.01, 95%CI of the first molar 7.12 to 26.85, 95%CI of the second molar 7.91 to 22.79) in the experimental model.

For the canine, the Fx and Mx values were negative, suggesting that the canine was applied a distal force during retraction. In the FE model, after partial thickening of the aligner at the posterior teeth, both Fx and Mx values increased, representing an increase in the retraction force on the canine. The decrease in the d value illustrated that the force was closer to the CR. Similarly, in the experimental model, the enhanced structure group exhibited higher Fx and Mx values compared to the control group but did not show statistical significance (*P*  > 0.05, 95%CI for Fx of canine −42.53 to 5.47, 95%CI for Mx of canine −4.75 × 10^2^ to 1.16 × 10^2^).

In summary, the finite element model and the experimental model showed similar trends. Changing the local thickness of the CA at the posterior teeth improved the biomechanics during retraction to prevent tipping movement of the posterior teeth.

### Force distribution of maxillary dentition

**Fig. 4:**
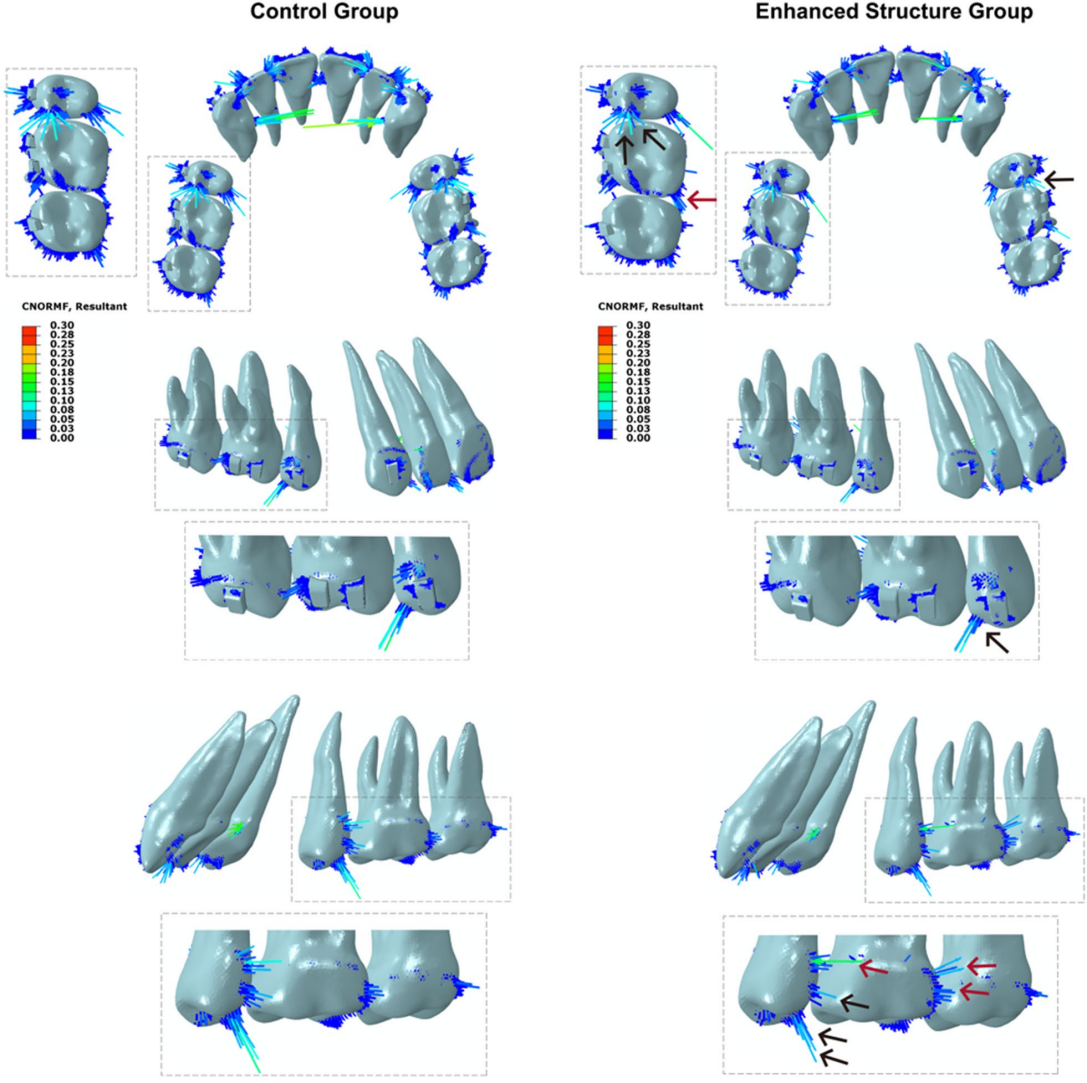
Force distribution of maxillary dentition (Unit: N): Colored lines represent forces. The color and length of the line reflect the force magnitude. Red arrow indicates an increase in force magnitude compared to the control group. Black arrow represents a decrease in force magnitude compared to the control group

As illustrated in Fig. 4, forces were predominantly concentrated on the anterior teeth and the distal side of the second premolar during anterior retraction. With the implementation of the enhanced structure of the posterior teeth, forces applied on the cervical third of the palatal surface of the second premolar and first molar relatively increased (red arrow), while forces applied on the occlusal third of the second premolar relatively decreased (black arrow). Consequently, for the second premolar, the force distribution was closer to the CR, reducing the risk of anchorage loss and increasing the likelihood of bodily movement of the tooth.

### Von-mises stress distribution of clear aligner

**Fig. 5:**
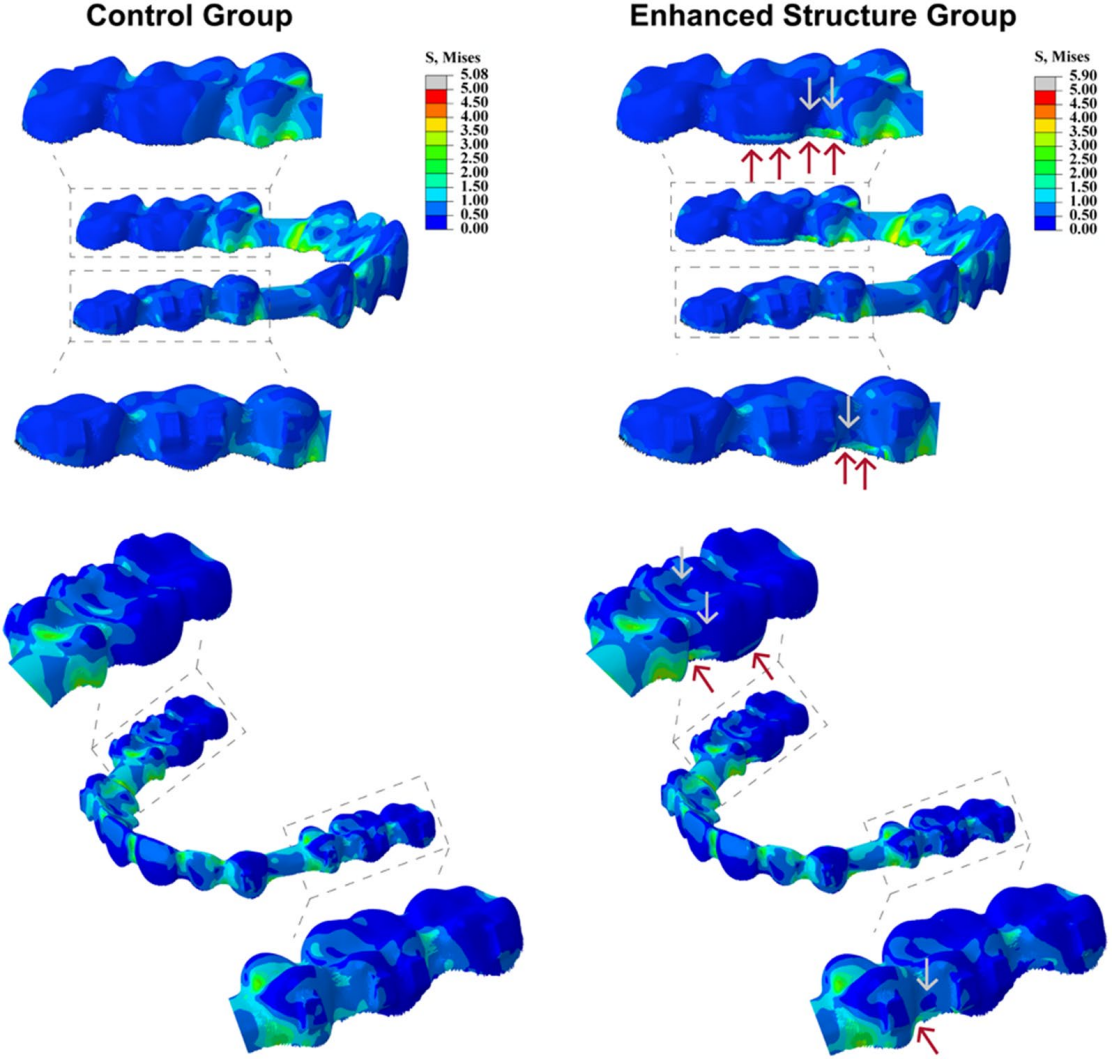
Von-mises stress distribution of clear aligner (Unit: MPa): Different colors reflect different stress levels. Red arrow indicates an increase in stress magnitude compared to the control group. Grey arrow represents a decrease in stress magnitude compared to the control group

The stress distribution of CA is illustrated in Fig. 5. In the control group, it can be seen that during the extraction space closure, the areas of higher stress were mainly located at the anterior teeth, the extraction space and the second premolar. After thickening of the buccal and palatal gingival sides of the posterior teeth, stress in the interproximal spaces between the posterior teeth corresponding to the thickened areas increased (red arrow), while the stress in the occlusal side of the second premolar and the first molar decreased (grey arrow). Since the enhanced structure was located at the gingival margin of the clear aligner, the stress was distributed closer to the CR of the premolar and molars, resulting in a reduction in the tipping movement of these teeth.

## Discussion

Anchorage control is critical for extracted patients with incisor protrusion or severe crowding. Loss of anchorage can be disastrous in these cases where strong anchorage is needed, so reinforcing anchorage of posterior teeth should be carried out throughout the orthodontic process. Our study proposed a new method of anchorage reinforcement in clear aligner therapy, which is to enhance the local stiffness by increasing the local thickness of CA, so that the CA can balance both the flexibility and the local stiffness, resulting in a reduction of mesial tipping of the posterior teeth. This new method is applicable during the entire process of orthodontics and has no impact on the design of the treatment staging. Localized thickening of the aligners can be performed during the fabrication phase without clinical operation and does not occupy chairside time. This approach is non-invasive and can be used in any case that requires reinforcement of anchorage. At the same time, it does not conflict with other ways of reinforcement anchorage and accordingly can be employed in combination with other alternatives. Consequently, this new approach of anchorage reinforcement has potential for clinical applications.

In this study, we observed that after partial thickening of the aligner at the posterior segment, the FE and experimental models both showed an increase in force magnitude on the molars, while a decrease in force magnitude was observed on the second premolar. Simultaneously, the force distribution in the posterior segment shifted toward the gingival side, closer to the CR of the tooth. This performance after partial thickening was more in accordance with optimal principles of biomechanics. Studies have shown that the area of PDL differs from tooth to tooth [31] and the size of a tooth’s PDL directly influences its resistance to movement and anchorage value [7]. The second premolar, being a single rooted tooth, has the smallest anchorage value among the posterior teeth and is located adjacent to the extraction space, and the tooth has a natural tendency to drift toward the extraction space where there is less resistance; therefore, it is very important to control the anchorage of the second premolar. The results of this study demonstrated that by thickening the aligners, the force exerted on the second premolar decreased by 18.65 g; while, the force distribution was closer to the CR of the tooth. This adjustment effectively reduces the mesial tipping of the posterior teeth and decreases the anchorage loss when closing the extraction space. Conversely, molars, being multi-rooted teeth, have anchorage values (53.3 mm^2^) that are twice as large as that of premolars (25.4 mm^2^) [32] and can withstand greater forces without mesial movement relative to premolars. While the first and second molars did experience an average increase in forces of 16.99 g and 15.35 g, respectively, due to the addition of the enhanced structure, this increased anchorage value effectively equipped the molars with the capacity to resist unwanted movement. The aforementioned phenomenon is beneficial for extraction cases to control the position of anchor teeth and partly avoid the “roller coaster effect”.

In fixed appliances, the sliding mechanics of space closure requires sufficiently stiff wires to prevent archwire bending, which can lead to the tipping of anchor teeth and the “roller coaster effect”. Burstone had proposed the idea that the posterior segment of the archwire needed to be more rigid than the anterior segment [18]. Drawing inspiration from this concept, we sought to enhance the stiffness of the posterior segment of CAs to facilitate space closure. Both the FE and experimental models demonstrated that this enhancement effectively reduced Fx and Mx values on the second premolar, and shifted force distribution closer to the gingival direction. There were two possible reasons for this outcome. Firstly, CAs exert orthodontic forces through elastic restoration. The counter diagram (Fig. 5) of the control group for FE model showed that the stress in the posterior segment of the aligner was mainly concentrated in the interproximal spaces of posterior teeth. Accordingly, when thickening the interproximal spaces of posterior teeth, the tensile stress generated in this section can be reinforced. Since the enhanced structure was located at the distal of the second premolar, it resulted in a rise in the distal force magnitude on the second premolar, reducing the tendency of mesial tipping of the second premolar and the possibility of anchorage loss. Secondly, the thickened site, positioned at the cervical third of the aligners, increased force near the gingival margin, shifting the action point of the resultant force toward the cervical, closer to the CR.

There are a variety of methods used to perform CA biomechanical studies, such as finite element analysis [33], photoelastic stress analysis [34] and micro-sensor [30]. In our study, two models, namely finite element analysis and experimental apparatus, were used to verify that partial thickening of CAs could indeed reduce the mesial tipping of anchor teeth in the process of anterior tooth retraction. Each model had its own advantages and complemented the other. Three-dimensional finite element analysis is commonly used to study the biomechanics of clear aligners, with the advantage of simulating the PDL as an anatomical structure [35]. However, in the FE model, the thickness of the aligners was even and the aligner was uniformly stretched when deformation occurred, unlike in actual clear aligners. Actual CAs are produced by vacuum thermoforming technology and do not have a uniform thickness on different tooth surfaces [16]. Therefore, they are not evenly tensile during deformation. To address this, our experimental model utilized realistic CAs to study the forces exerted on each tooth, compensating for the limitations of the FE model. Although the experimental model lacked in vitro PDL representation, it effectively explored the biomechanics in orthodontic appliances with uneven thickness. Admittedly, due to the inherent differences between these two models, a direct comparison of measured values between the two models was not available. We observed the same trends of the variation in force magnitude of the two models separately after adding the enhanced structure, and thus drew the corresponding conclusions. Therefore, these two approaches complemented each other and collectively demonstrated a tendency for the enhanced structure to reinforce the anchorage of posterior teeth.

Orthodontic forces are classified as light, moderate and heavy forces based on their magnitude. The use of light but lasting force is preferred in clinical practice as it allows for rapid tooth movement while minimizing root resorption. Excessive orthodontic force can lead to ischemic necrosis and hyalinization in the PDL, leading to undermining resorption and slowed tooth movement [7]. Our results from the FE model indicated a maximum force of approximately 250 g, which exceeds the range of light forces. This is due to the fact that the FE model simulates the initial forces generated by the aligners; while, the force values of the aligners decay rapidly by half or more after initial insertion due to stress relaxation [36]. Consequently, stable orthodontic forces exerted by CAs are considered light forces. Despite the initially higher stress value, studies have shown that CAs have a lower risk of root resorption than fixed appliances due to the stress relaxation and intermittent loading method [37, 38]. In the experimental model, the measured F and M values were twice as large as those in the FE model due to the absence of the PDL as a physiological structure in the experimental apparatus. The PDL has a cushioning effect on forces applied to teeth, accommodating forces exerted on the crown [39]. In contrast, the experimental model is mechanically connected to the sensor by screws, which are much stiffer than the PDL. Therefore, this inherent difference between the models leads the experimental setup to record much larger force values than the FE model that incorporates the PDL.

Moment is generated by a force acting at a certain distance, causing the tooth to have a tendency to rotate around the CR. Quantitatively, it is the multiplication of the force and the perpendicular distance from the point of force application to the CR. As a result, the error in moment value is magnified compared to the error in force value, which is noticeable in the experimental model, and a similar phenomenon has been reported in the previous study [27]. This is likely due to the fixed mechanical connection between the sensor and the resin tooth of the experimental device, and the absence of the PDL, which is where this experimental model still needs to be improved. Therefore, in our study, we observed the distance change through the FE model (Figs. 3C, 4 and 5) instead of calculating it directly in the experimental model.

## Limitations

Although finite element analysis is one of the best ways to analyze biomechanics delivered by orthodontic appliances, it still has its limitations in accurately simulating the true oral environment (like body temperature and saliva), and it cannot simulate the continuous application of orthodontic forces. Moreover, the experimental model, despite using real CAs, is difficult to mimic the PDL in an in vitro setting. Therefore, further optimization in model construction is needed in future studies. Additionally, the efficacy of this enhanced structure proposed in our study requires validation through clinical applications. Nevertheless, our study presents an innovative approach to address the “roller coaster phenomenon” and reinforce the anchorage of posterior teeth when closing the extraction space using CAs.

## Conclusions

Our study, by both the FE model and the experimental model, indicated that the biomechanics of the clear aligner during space closure was optimized by locally thickening the posterior segment of the aligner to form the enhanced structure. The enhanced structure resulted in a decrease in the force magnitude on the second premolar and allowed the force distribution closer to the CR, therefore reducing the mesial tipping of anchor tooth and mitigating the roller coaster effect related to orthodontic extraction cases. Consequently, our study provided a new dimension for anchorage reinforcement in clear aligner therapy.

### Supplementary Information

**Additional file 1**. Supplementary Figures 1-3 and Supplementary Tables 1-6.

## Data Availability

Data will be made available on reasonable request.

## Abbreviations

CA: Clear aligner
CR: Center of resistance
CBCT: Cone-beam computed tomography
PDL: Periodontal ligament
FE: Finite element
LCSYS: Local coordinate system
F: Force
M: Moment
d: Distance
