# Supplementary material for: The effect of enhanced structure in the posterior segment of clear aligners during anterior retraction: a three-dimensional finite element and experimental model analysis
Source: Prog Orthod. 2024 Jan 15;25:3. doi: 10.1186/s40510-023-00502-2 (PMC10788328; doi:10.1186/s40510-023-00502-2)
Supplement: Supplementary file 1 — Additional file 1. Supplementary Figures 1-3 and Supplementary Tables 1-6. [file 40510_2023_502_MOESM1_ESM.docx]

**Supplementary Information**

**for**

**The Effect of Enhanced Structure in the Posterior Segment of Clear Aligners during Anterior Retraction: A Three-Dimensional Finite Element and Experimental Model Analysis**

**This file includes:**

Supplementary Figures 1-3

Supplementary Tables 1-6


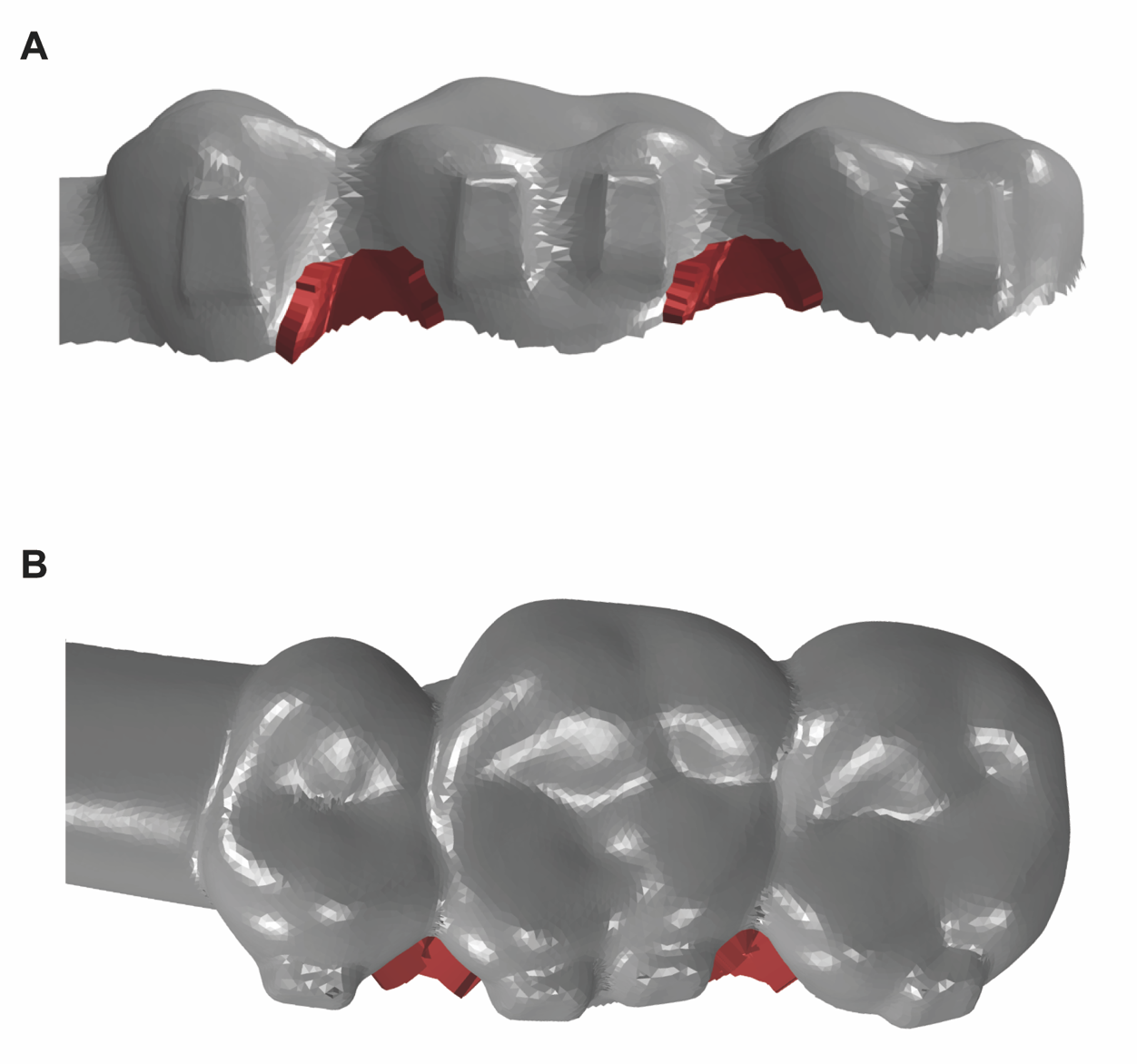


**Supplementary Figure 1**: Buccal enhanced structure positioned at buccal interproximal space between the posterior teeth: **A**, Buccal view; **B**, Occlusal view.


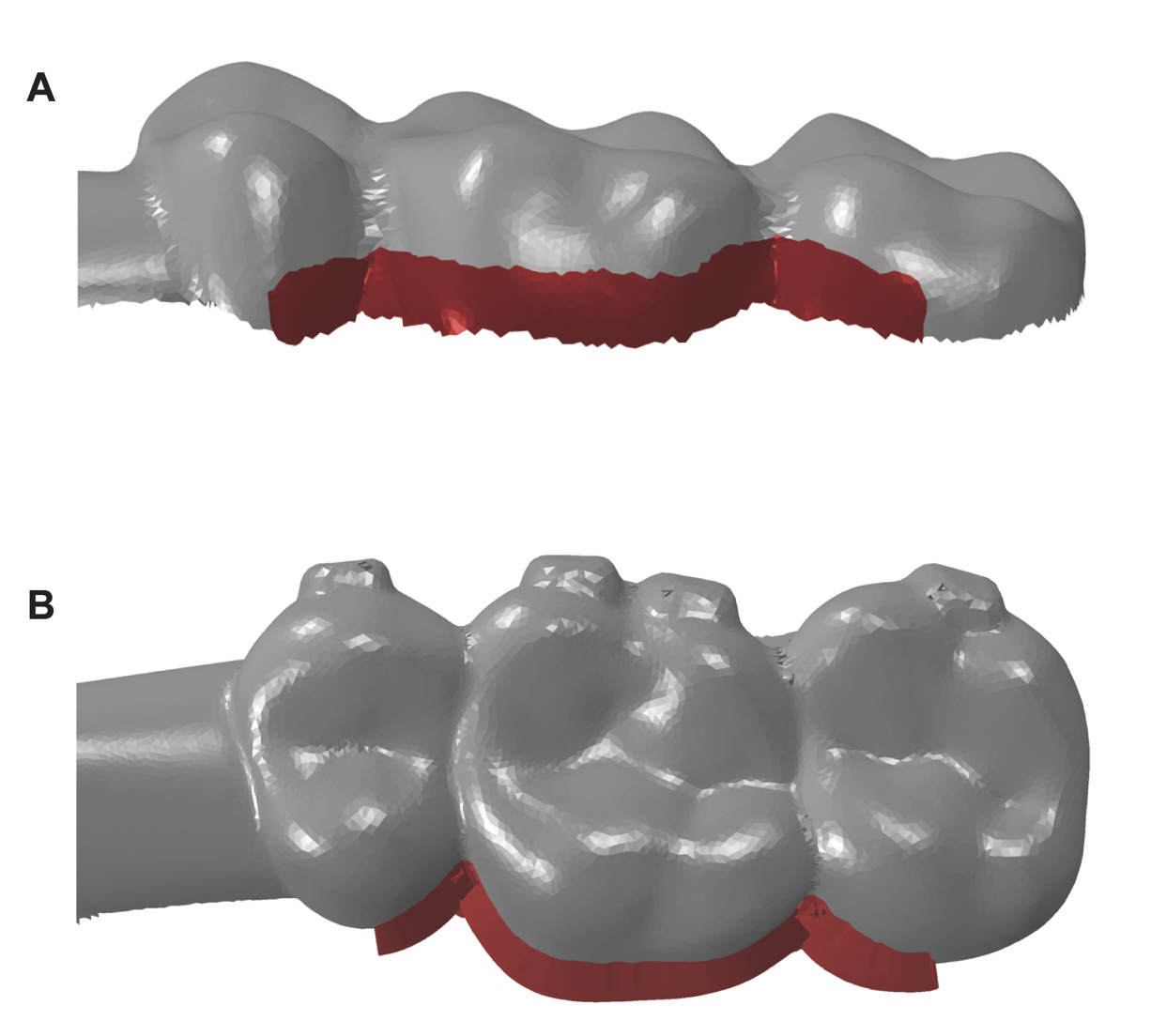


**Supplementary Figure 2**: Lingual enhanced structure positioned at linguocervical ridge of posterior teeth: **A**, Palatal view; **B**, Occlusal view.


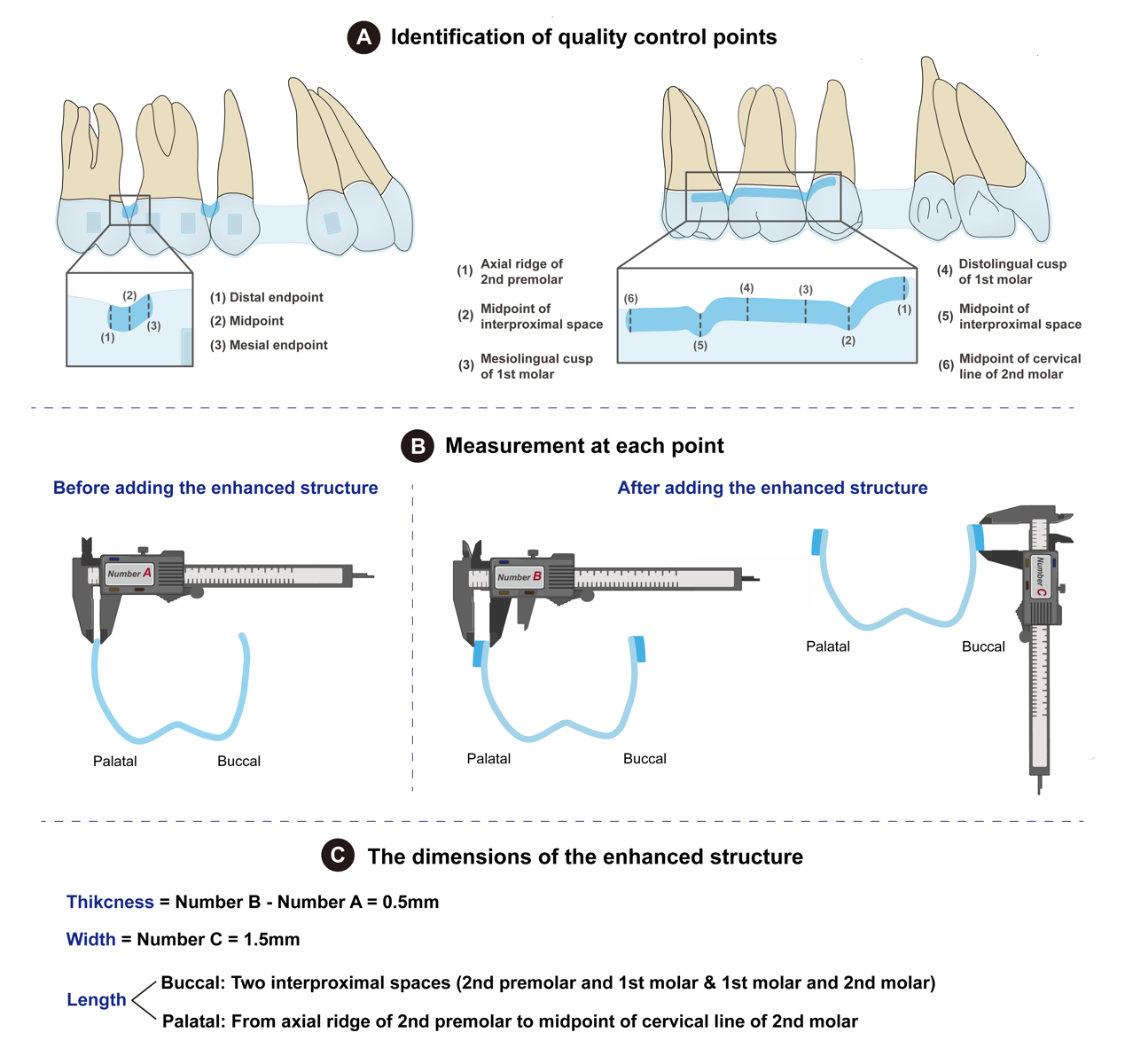


**Supplementary Figure 3**: Quality control approach for enhanced structures: **A**, Identification of quality control points; **B**, Measurement Method; **C**, The dimensions of the enhanced structure.

**Supplementary Table 1:** Forces and moments in the local coordinate system (LCSYS) of the finite element model for buccal enhanced structure. (Unit: 10^2^ g)

|  | **Fx** | **Fy** | **Fz** | **Mx** | **My** | **Mz** |
| --- | --- | --- | --- | --- | --- | --- |
| **17** | 0.9196 | 0.1546 | -0.7172 | 6.3250 | 3.0585 | 1.0301 |
| **16** | 1.5582 | 0.3397 | -0.0718 | 7.0188 | 3.4866 | 4.9588 |
| **15** | 1.4489 | 0.4441 | 0.6484 | 7.3384 | 1.2097 | 0.8686 |
| **13** | -2.4957 | -0.1821 | -0.2223 | -22.7975 | -1.4694 | -1.5916 |
| **12** | -0.0319 | 0.6442 | 1.4124 | -4.2967 | 7.0357 | -0.5096 |
| **11** | -0.0646 | 0.7011 | 0.4306 | -1.9231 | 9.6023 | -0.7836 |
| **21** | -0.1578 | 0.6795 | 0.4074 | -2.5451 | 9.6109 | 0.7914 |
| **22** | -0.0857 | 0.6226 | 1.3934 | -4.7525 | 6.8704 | 0.4945 |
| **23** | -2.3197 | -0.1344 | -0.2293 | -22.5491 | -1.4765 | 2.5905 |
| **25** | 1.4986 | 0.4430 | 0.5508 | 7.3240 | 1.3011 | -0.7586 |
| **26** | 1.4961 | 0.3711 | 0.0078 | 6.8652 | 3.5593 | -5.1391 |
| **27** | 0.9409 | 0.1288 | -0.7555 | 6.1691 | 2.6451 | -1.1289 |

**Supplementary Table 2:** Forces and moments in the LCSYS of the finite element model for lingual enhanced structure.

|  | **Fx** | **Fy** | **Fz** | **Mx** | **My** | **Mz** |
| --- | --- | --- | --- | --- | --- | --- |
| **17** | 0.9510 | 0.1895 | -0.7029 | 6.2799 | 2.9017 | 0.7303 |
| **16** | 1.5616 | 0.2799 | -0.1063 | 7.1806 | 3.0488 | 3.7895 |
| **15** | 1.4556 | 0.4078 | 0.6335 | 7.1760 | 0.9216 | 1.0632 |
| **13** | -2.5061 | -0.1786 | -0.1911 | -23.0589 | -1.3314 | -1.5596 |
| **12** | -0.0352 | 0.6525 | 1.4364 | -4.3766 | 7.1570 | -0.5119 |
| **11** | -0.0684 | 0.7093 | 0.4423 | -1.9661 | 9.7534 | -0.8022 |
| **21** | -0.1603 | 0.6881 | 0.4190 | -2.5525 | 9.7552 | 0.8047 |
| **22** | -0.0858 | 0.6296 | 1.4135 | -4.7975 | 6.9773 | 0.4892 |
| **23** | -2.3197 | -0.1338 | -0.2044 | -22.7359 | -1.3646 | 2.5393 |
| **25** | 1.5275 | 0.3986 | 0.5289 | 7.2654 | 0.9856 | -0.8992 |
| **26** | 1.4585 | 0.3003 | -0.0335 | 7.0713 | 3.0934 | -4.2791 |
| **27** | 0.9767 | 0.1742 | -0.7208 | 6.1858 | 2.5141 | -0.5947 |

**Supplementary Table 3**: Forces and moments in the LCSYS of the finite element model for linguobuccal enhanced structure.

|  | **Fx** | **Fy** | **Fz** | **Mx** | **My** | **Mz** |
| --- | --- | --- | --- | --- | --- | --- |
| **17** | 0.9395 | 0.1734 | -0.7008 | 6.3647 | 2.9881 | 0.8332 |
| **16** | 1.6106 | 0.3138 | -0.0936 | 7.1036 | 3.2616 | 4.3500 |
| **15** | 1.4275 | 0.4263 | 0.6142 | 7.3185 | 0.9920 | 0.9405 |
| **13** | -2.5108 | -0.1818 | -0.1938 | -23.0177 | -1.3935 | -1.5451 |
| **12** | -0.0355 | 0.6507 | 1.4348 | -4.3711 | 7.1365 | -0.5037 |
| **11** | -0.0674 | 0.7111 | 0.4421 | -1.9644 | 9.7556 | -0.7932 |
| **21** | -0.1600 | 0.6887 | 0.4203 | -2.5645 | 9.7579 | 0.7969 |
| **22** | -0.0881 | 0.6281 | 1.4146 | -4.8127 | 6.9601 | 0.4844 |
| **23** | -2.3288 | -0.1340 | -0.2040 | -22.7407 | -1.4278 | 2.5358 |
| **25** | 1.4908 | 0.4250 | 0.5175 | 7.2688 | 1.0875 | -0.8364 |
| **26** | 1.5052 | 0.3305 | -0.0283 | 7.0081 | 3.3391 | -4.7604 |
| **27** | 0.9821 | 0.1557 | -0.7222 | 6.2684 | 2.5694 | -0.7205 |

**Supplementary Table 4**: Forces and moments in the LCSYS of the finite element model for control group.

|  | **Fx** | **Fy** | **Fz** | **Mx** | **My** | **Mz** |
| --- | --- | --- | --- | --- | --- | --- |
| **17** | 0.9170 | 0.1721 | -0.6786 | 6.2751 | 2.8387 | 0.9818 |
| **16** | 1.4538 | 0.3065 | -0.0610 | 7.2769 | 3.2651 | 4.4949 |
| **15** | 1.4509 | 0.4044 | 0.6322 | 7.4608 | 1.1961 | 1.0400 |
| **13** | -2.4237 | -0.1682 | -0.2290 | -22.3040 | -1.4000 | -1.6194 |
| **12** | -0.0360 | 0.6340 | 1.3724 | -4.0847 | 6.9780 | -0.5264 |
| **11** | -0.0634 | 0.6901 | 0.4169 | -1.8744 | 9.4071 | -0.7882 |
| **21** | -0.1571 | 0.6667 | 0.3928 | -2.4623 | 9.4344 | 0.7964 |
| **22** | -0.0868 | 0.6147 | 1.3528 | -4.5312 | 6.8075 | 0.5122 |
| **23** | -2.2586 | -0.1179 | -0.2293 | -21.9616 | -1.3978 | 2.6322 |
| **25** | 1.5060 | 0.4036 | 0.5468 | 7.4548 | 1.3127 | -0.9686 |
| **26** | 1.4018 | 0.3340 | 0.0153 | 7.0609 | 3.3008 | -4.7409 |
| **27** | 0.9321 | 0.1502 | -0.7204 | 6.1617 | 2.4479 | -0.9568 |

**Supplementary Table 5**: Forces and moments in the LCSYS of the experimental model for linguobuccal enhanced structure.

|  | **Fx** | **Fy** | **Fz** | **Mx** | **My** | **Mz** |
| --- | --- | --- | --- | --- | --- | --- |
| **17** | 3.0739±0.3105 | -0.4168±0.1352 | -0.1028±0.3621 | 29.5130±4.6369 | -5.4495±2.3296 | -0.9158±1.8643 |
| **16** | 3.5572±0.3971 | 1.9456±0.2221 | -0.4265±0.2987 | 29.1242±3.0170 | 15.9672±2.8310 | 20.0538±2.1614 |
| **15** | 2.5305±0.2361 | 0.7690±0.0849 | 1.3686±0.4297 | 22.6496±2.5601 | 5.2639±0.9060 | 2.6678±0.8033 |
| **13** | -4.6719±0.3627 | 0.7054±1.0776 | 0.1694±0.1852 | -45.1050±5.1009 | 17.0049±8.0234 | 5.2855±4.8257 |
| **12** | 0.3380±0.1038 | 2.1655±0.0945 | 0.9356±0.1144 | 3.4273±1.4889 | 26.449±0.9905 | 0.4750±0.7161 |
| **11** | -0.5597±0.2220 | 1.0938±0.3480 | 2.1277±1.0386 | -5.4781±2.2517 | 16.4295±5.0315 | 2.2489±0.6868 |
| **21** | -0.2051±0.2576 | 2.2203±0.6539 | 0.9827±0.1747 | -1.2684±2.5652 | 30.5595±8.3753 | 1.8115±1.0940 |
| **22** | 0.1606±0.2765 | 2.4760±0.4880 | 1.4665±0.3530 | 2.7822±2.4448 | 23.4827±25.2192 | 0.4445±0.2098 |
| **23** | -4.6415±0.2413 | 0.6045±0.8457 | 0.5981±0.5513 | -47.6627±4.2949 | 5.1657±8.7971 | 9.7772±2.6258 |
| **25** | 2.1577±0.2979 | 0.6273±0.1341 | 1.4425±0.5885 | 18.4593±1.4317 | 2.6370±2.7360 | 4.1302±3.2299 |
| **26** | 3.6257±0.6125 | 0.6666±0.4052 | -1.1609±0.4162 | 30.8120±6.3315 | 28.1222±21.1654 | -26.3139±10.6757 |
| **27** | 2.3247±0.3436 | 0.9461±0.1882 | -0.3467±0.3000 | 36.0088±4.3013 | 18.0657±7.9873 | -22.1582±3.7878 |

**Supplementary Table 6**: Forces and moments in the LCSYS of the experimental model for control group.

|  | **Fx** | **Fy** | **Fz** | **Mx** | **My** | **Mz** |
| --- | --- | --- | --- | --- | --- | --- |
| **17** | 2.9205±0.2837 | -0.3635±0.1388 | -0.1707±0.3415 | 28.1286±4.3475 | -4.7535±2.3365 | -0.9047±1.9665 |
| **16** | 3.3873±0.3603 | 1.8022±0.2303 | -0.3379±0.3010 | 28.0129±2.5813 | 14.3240±2.1692 | 18.9483±1.8876 |
| **15** | 2.7170±0.2397 | 0.7554±0.1494 | 1.3017±0.4350 | 22.8506±2.8426 | 3.6000±1.0076 | 2.4687±0.7018 |
| **13** | -4.4866±0.4482 | 0.6767±1.0823 | 0.2791±0.2582 | -43.3081±2.8098 | 18.3277±10.0816 | 6.1332±3.9684 |
| **12** | 0.3545±0.1350 | 2.1424±0.1320 | 0.9002±0.1225 | 3.7037±1.8201 | 26.1173±1.5481 | 0.4831±0.6649 |
| **11** | -0.5825±0.2139 | 1.0411±0.3588 | 2.1007±1.1631 | -5.2272±3.2036 | 15.7427±5.5008 | 2.0443±0.8912 |
| **21** | -0.1973±0.2596 | 2.1929±0.6535 | 0.9731±0.2116 | -1.4209±2.5221 | 30.0081±8.5249 | 1.6308±0.8764 |
| **22** | 0.1484±0.2866 | 2.4537±0.5025 | 1.4696±0.4706 | 2.6665±2.8184 | 31.9591±6.5790 | 0.3522±0.2691 |
| **23** | -4.5103±0.2687 | 0.6282±0.8204 | 0.5595±0.5399 | -46.7189±3.4802 | 3.1753±9.8895 | 9.8804±4.8928 |
| **25** | 2.3357±0.3813 | 0.6495±0.1616 | 1.5138±0.6389 | 17.5735±1.5860 | 3.5958±4.2928 | 4.0226±2.2533 |
| **26** | 3.5332±0.5699 | 0.7170±0.5131 | -1.0489±0.3575 | 30.1938±6.6793 | 25.9076±22.6136 | -23.3989±11.1060 |
| **27** | 2.1846±0.3094 | 0.8817±0.2009 | -0.3901±0.2813 | 34.4948±3.6039 | 16.1975±7.6236 | -20.1833±4.0401 |
